# The effectiveness of Doppler controlled hemorrhoidal artery ligation based on preliminaries results

**DOI:** 10.11604/pamj.2013.15.159.2190

**Published:** 2013-08-31

**Authors:** Rajae Yamoul, Gilles Attolou, Noureddine Njoumi, Siffedine Alkandry, Moulay El Hassane Tahiri

**Affiliations:** 1 Department of Visceral Surgery and proctology, HMIMV, Rabat, Morocco

**Keywords:** Symptomatic hemorrhoids, Hemorrhoidal Artery Ligation, Doppler, mucopexy

## Abstract

In this work, we discuss the preliminary results of the effectiveness of the hemorrhoidal artery ligation under control Doppler as a new technique for the treatment of hemorrhoids. We report the results of patients with hemorrhoids we have followed over a period of one year who were treated with HAL Doppler. The intra-and postoperative complications were monitored. Patient follow-up was established on the immediate postoperative period, after one month, six months and one year of evolution. Monitoring parameters were both objective (prolapse) and subjective (pain, discomfort, bleeding, satisfaction). 120 patients, all stages combined, were treated with HAL Doppler. The pain is easily controlled with painkillers. Residual rectal bleeding was noted in 3% of the cases. In addition, there was one case of recurrent prolapse which underwent reoperation. After one month, 86,5% patients were satisfied. The hemorrhoidal artery ligation under control Doppler is an easy technique, well accepted by patients who are increasingly demanding it. It is less invasive, less painful and gives fewer complications. It is not only effective for stage III and IV hemorrhoids for which the effect is spectacular but also for stages I and II symptoms, which can expand its indications.

## Introduction

Hemorrhoidal disease is a common reason for consultation in proctology and a public health problem responsible for discomfort and an urgent request relief [1]. The patient often consults for rectal bleeding, or pain.

Many techiques are available and described in the literature to treat this diseases [2]. However, there is no treatment “Gold Standard”. The consensus is partly based on the fact that the indication for surgery depends on the severity of the symptoms which remain subjective but also on the choice of treatment which is based on the grade of hemorrhoids and the local experience of the team [3–5].

Currently, combining an anuscope and a Doppler transducer, makes it possible to detect and bind the arteries responsible for congestion selectively thereby to reduce the blood flow and thus collapsing hemorrhoids. This approach is a new technique that has revolutionized hemorrhoidal surgery. The aim of this work is to present the preliminary results of this technique from 120 cases treated by HAL Doppler.

## Methods

This study includes the results of 120 patients who underwent hemorrhoidal artery ligation including 88 men and 32 women. The average age was 51 years with extremes of 21 and 76 years; All patients operated were symptomatics (Table 1). 111 patients were treated medically, 2 underwent instrumental treatment (band ligation), and 05 of which 04 were operated by the technique of Milligan and one by the Longo technique. Clinical examination found stage IV hemorrhoids in 55 cases (45, 83%), stage III in 42 cases (35%) stage II in 20 cases (16.6%), stage I in 03 cases (2.5%). Stages I and II operated were symptomatic.

**Table 1:** Different hemorrhoidal symptoms

The symptom	n
bleeding	83
Pain	76
Prolapse	97
discomfort	120

All patients received anorectal preparation the night before and a few hours before the procedure. They were operated under spinal anesthesia in 117 cases, and general anesthesia in 2 cases, a local anesthesia was efficient for one patient. They were operated in a size position, after their consent and after clear information explaining the nature of the planned surgical procedure.

**Figure 1:**
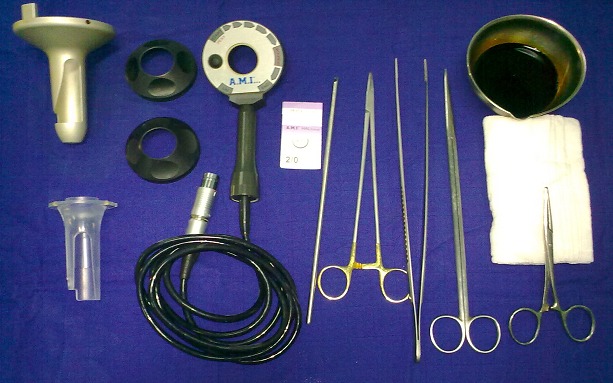
The material used for hemorrhoidal artery ligation under control Doppler

**Figure 2:**
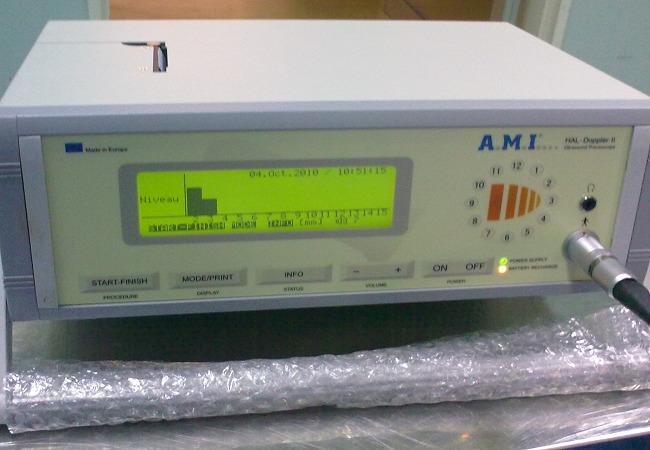
The branch hemorrhoidal to bind is detected by a sensor, which results in a sound signal and an image on the monitor indicating the depth of the artery

**Figure 3:**
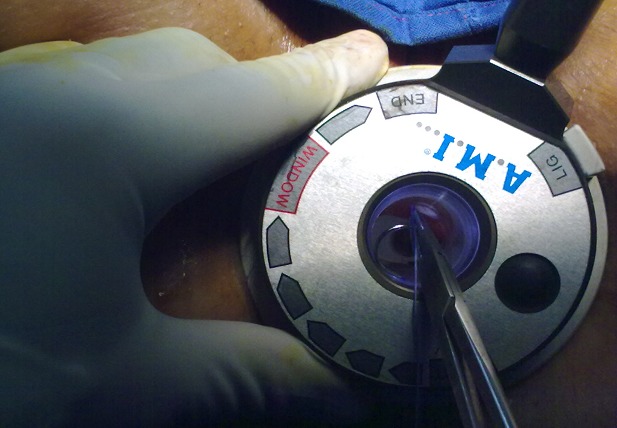
Once it is detected, the artery is linked through the window in the anuscope

We used an anuscope with a Doppler transducer included in the wall thereof for detecting the signal of hemorrhoidal arteries using a disposable probe (Figure 1, Figure 2), then bound by a point X in yarn 2/0 absorbable provided with a needle of a curvature of 5/8 via a side window in the anuscope specially equipped and located above the transducer (Figure 3).

**Figure 4:**
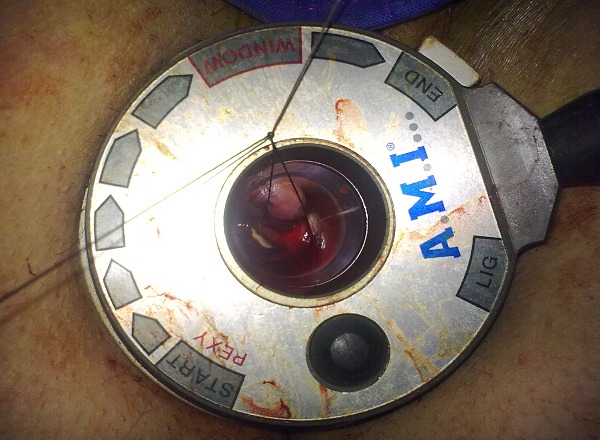
Mucopexy involves lifting and securing the hemorrhoidal prolapse into the anal canal

**Figure 5:**
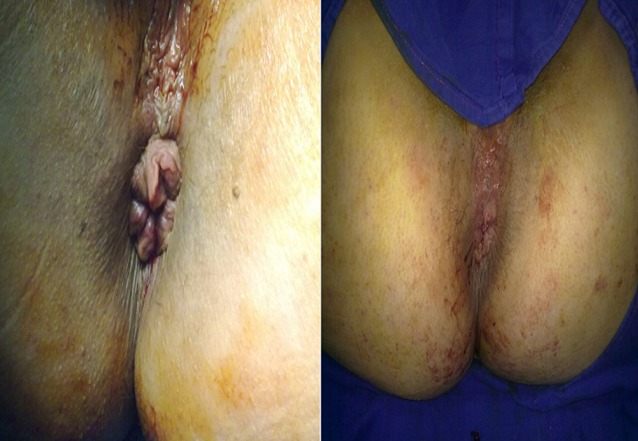
Photos taken before (A) and after (B) ligation and mucopexy

Many ligatures are then performed on the circumference of the lower rectum without preferential localization at two levels spaced apart from each other by about twenty millimeters. Making the second exploration also over the entire circumference, after adding a specific ring on the basis of the anuscope. The decrease in the Doppler signal confirmed the effectiveness of ligatures. Each time there was a prolapse, a mucopexy was systematic (Figure 4, Figure 5).

## Results

The average duration of surgery was 28 min with a range of 9 min and 76 min. Vascular ligations are performed at two successive levels; their average number is 8 ligatures with extremes of 02 and 14 ligatures by intervention. A mucopexy (Recto Anal Repair: RAR) is performed in 97 patients, a fissurectomy in 02 cases, lateral internal sphincterotomy in 06 cases, with a fistulectomy in 02 cases. No intra operative complications were noted.

The mean duration of hospitalization was 24 hours. Two patients were discharged from the hospital the same day of surgery, 96 after 24 hours, 17 after 02 days, and 05 after 03 days. The effectiveness of the technique has been tried in the immediate postoperative period, at J + 1, the 1rst month, the 3rd month, the 6th month and at the first year on subjective parameters (pain, satisfaction, bleeding) and objectives (rectal bleeding, prolapse).

Pain was assessed subjectively by the first postoperative day visual analog scale (VAS). 19 patients felt no pain. In other cases the pain intensity on D +1 ranged as follows: VAS 3 in 43 cases, 45 cases in four VAS, VAS 6 in 13 cases.

**Figure 6:**
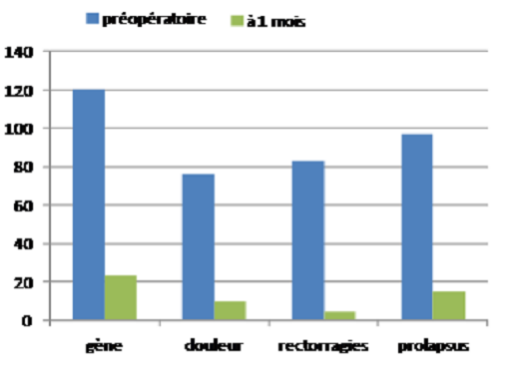
One month later the intervention, there was a net decline of symptoms in many patients

The pain was easily controlled with painkillers level I in the 62 cases, 45 cases used the Level II painkillers and painkillers level III were used only in 03 cases. After a month of surgery, only 10 patients (12%) have maintained the pain which became less intense (Figure 6).

After a month of surgery, only 10 patients (12%) have maintained the pain which became less intense (Figure 6). Of the 83 patients operated on for bleeding hemorrhoids, 4 still had bleeding but still minimal.

In addition, 15 patients had residual prolapse. They were re-operated by including a technique LONGO. Another patient was re-operated after a year for a second offense, In terms of personal satisfaction after one month, 73% of patients were satisfied, 13.5% were very satisfied and 13.5% were unhappy.

During follow-up of patients operated, 5 have developed an anal fissure of which 3 were treated surgically, and there was one case of postoperative thrombosis.

## Discussion

Hemorrhoids are normal vascular formations. We distinguish between external hemorrhoids, which are dependent on the hemorrhoidal artery and lower subcutaneous location are under-pectineal, and internal hemorrhoids located above the dentate line, which arise from the superior hemorrhoidal artery which gives three branches: left, right anterior and posterior.

Vascular and mechanical factors impair these hemorrhoids and are responsible for an imbalance that increases arterial irrigation and decreases venous drainage which is manifested clinically by rectal bleeding, pain or prolapsed [3]. It is important to remember that the hemorrhoidal syndrome is a benign disease and its treatment therefore must be harmless as possible.

Several therapeutic modalities are described in the literature; the most recent is a hemorrhoidal artery ligation. Historically, it is a Japanese surgeon, Kazumasa Morinaga, who had the idea of this technique in 1995, the principle is to identify and link in Doppler arterial branches that travel in the direction of the rectal wall hemorrhoidal internal network in order to relieve the internal hemorrhoidal tissue [6–8], its effectiveness is verified by the decrease in the Doppler signal. The procedure is usually done under spinal anesthesia, but it can be done under local anesthesia [7]. The average duration of the gesture is around about thirty minutes.

Outside the external hemorrhoidal pathology (thrombosis and skin tags) that do not seem to be a good indication of the hemorrhoidal artery ligation, it is now more appropriate for any internal hemorrhoidal disease at any stage. Indeed, it is effective in 85% of cases of bleeding hemorrhoids, and in 75% of cases of prolapse and allows in 70% of cases the relief of pain due to congestion hemorrhoidal [9–15] which confirms our preliminary results with 85% pain relief, 87% stop rectal bleeding, 94% reduction of the prolapse, and 86% of satisfaction.

Good tolerance is consistent across all series. Postoperative pain are seen in 10-55% of cases, however, they are easily relieved by analgesics bearing and are quickly resolve [7, 13]. Bleeding, including eschar falls have been reported in 1-12% of cases depending on the series [7, 9, 10, 14, 15], hemorrhoidal thrombosis have been reported in 5% of cases [8, 11, 13, 14].

In addition, this technique has the advantage of a very short hospitalization or outpatient [9, 10] a rapid recovery of the activity [10–13] and respect the anatomy of the anal canal is thus no stenosis or incontinence has been described so far. In short, the hemorrhoidal artery ligation under control Doppler technique is attractive for several reasons: first its interesting concept, speed and ease of implementation, its side effects virtually zero and its effectiveness especially undeniable

However, although it is a known technique for 15 years, it is not yet common practice. On the other hand, there are questions about the long-term results and the actual existence of a superiority of this technique compared to other. In terms of recurrence and patient satisfaction in the long term, the results vary according to the published series [7, 8, 12, 15].

For an average follow up one year recurrence of bleeding are seen in less than 3% of cases [7]; the recurrence rate was even higher when it comes to stage IV hemorrhoids [10, 13, 14] which led to perfect the technique by performing, in addition to arterial ligation, a “mucopexy” localized using a longitudinal suture in the mucosa of the lower rectum, with regard to hemorrhoids prolapse which seemed important to them [13], leading some authors to associate now this mucopexy each time it is stage IV prolapsed.

## Conclusion

Hemorrhoidal artery ligation under Doppler control is an innovation that began to spread and remains attractive, its concept is interesting and definitely effective; however many questions need answer particular its place in the therapeutic strategy of hemorrhoidal disease compared to other techniques. Its cost remains relatively high in our context which limits the availability of this technology to the entire population.
